# Real-world Follow-up Practice of Children With Coeliac Disease: A Cross-sectional Study From Western Sweden

**DOI:** 10.1097/PG9.0000000000000191

**Published:** 2022-03-17

**Authors:** Jennie Oskarsson, Anna Myleus, Karl Mårild

**Affiliations:** From the * Bräcke Diakoni outpatient clinic, Alingsås, Sweden; † Department of Public Health and Clinical Medicine, Family Medicine, Umeå University, Umeå, Sweden; ‡ Department of Paediatrics, Institute of Clinical Sciences, Sahlgrenska Academy, Gothenburg, Sweden; § Department of Paediatric Gastroenterology, Queen Silvia Children’s Hospital, Gothenburg, Sweden

**Keywords:** celiac disease, children, follow-up, adherence

## Abstract

Coeliac disease (CD) is one of the most common chronic diseases of childhood. Follow-up of CD aims to ensure dietary adherence and prevent disease complications, but there are few real-world data on how its management in children is conducted. This study aimed to survey the follow-up practice of pediatric CD in Western Sweden. Two web-based surveys were distributed to all 22 pediatric outpatient clinics rendering answers from 48 physicians and 12 dietitians. Overall, clinical practice was similar throughout the region and in line with national and international CD guidelines, including an annual to biannually follow-up frequency and dietary adherence assessment through unstructured interviewing and serology measurements. The study identified possible areas of improvement, such as implementing a formal transition process to adult care and the use of validated questionaries to assess dietary adherence. Additionally, a positive attitude towards electronic-health technologies (eHealth) as part of CD follow-up was identified.

**What Is Known**

- Coeliac disease (CD) is among the most common chronic disease of childhood.
- Follow-up of CD aims to ensure a strict gluten-free diet and monitor disease complications.
- Real-world data on the performance of CD follow-up care in children are scarce.

**What Is New**

- Through a region-wide survey of pediatricians and dieticians caring for CD, we report the follow-up practice of pediatric CD in Western Sweden.
- CD was followed annually-biannually, including CD-serology tests and dietary adherence assessment by unstructured interviewing.
- Opportunities for quality improvement included formal transition to adult care, validated dietary adherence questionaries, and increased educational activities on CD.
- Positive attitudes towards electronic-health technologies and an expanded role of the dietitian were indicated.

Coeliac disease (CD) is one of the most common chronic diseases among children in Sweden (1), and elsewhere (2). In CD, gluten ingestion in genetically susceptible individuals causes small-intestinal villus atrophy, and raised anti-tissue transglutaminase antibodies (TTG) (3). The disease is associated with various intestinal and extra-intestinal manifestations, including impaired growth and quality of life (1,4). Although a strict gluten-free diet (GFD) can effectively alleviate symptoms and achieve mucosal healing, such diet is cumbersome and non-adherence common, particularly among adolescents (5).

Patients with CD are recommended regular follow-up to ensure dietary adherence and prevent disease complications (6), but there are few real-world data on how and at what frequency follow-up care is performed in children with CD (7–9). There is also limited understanding on how dietitian counseling in pediatric CD is actually conducted. Data from a real-world setting are fundamental to identify areas of quality improvements in the care of pediatric CD.

This study aimed to survey how CD follow-up was conducted for children living in the Western part of Sweden (region of Västra Götaland, VGR).

## METHODS

We report the follow-up practice of children with CD based on 2 web-based surveys administrated to all 22 general pediatric outpatient clinics in VGR.

### Setting

There are approximately 1.7 million inhabitants – a sixth of the Swedish population – living in VGR. Each of the 22 pediatric outpatient clinics of VGR is, based on their geographically location, divided into four groups and affiliated with a pediatric inpatient clinic of that area (See Supplemental Digital Content 1 Figure, http://links.lww.com/PG9/A76, which shows a map of all pediatric outpatient clinics). After being diagnosed at a pediatric inpatient clinic, children with CD are referred to one of the 22 pediatric outpatient clinics for follow-up by both a pediatrician and dietician. All clinics had access to a designated dietitian for nutritional support of listed patients.

The number of celiac patients cared for by each clinic differs according to the size of the clinic. For example, 113 doctor’s visits related to CD follow-up were held in 2020 at the pediatric outpatient clinic in Hisingen, constituting one of the largest clinics in the survey; in contrast, Alingsås pediatric outpatient clinic (one of the smallest clinics that participated) had 35 doctor’s visits for CD in 2020.

### Web-based Survey

All head managers of each pediatric outpatient clinic were contacted to distribute a web-based survey to all permanently employed pediatricians and dietitians at the clinic; while similar, different surveys were used for pediatricians and dietitians to capture follow-up practices that may be specific for each of these personnel categories. In this study, follow-up care will be referred to as “pediatrician-led” when provided by a pediatrician and “dietician-led” when provided by a dietician. Data were collected between January and February 2021 using Microsoft Forms (Microsoft, Redmond, WA).

#### Survey of Pediatrician-led Follow-up

We used 52 questions to survey the timing and frequency of follow-up as well as how the follow-up care was conducted, including methods used to monitor dietary adherence, symptoms, growth, nutritional status, and comorbidities (See Supplemental Digital Content 2 Table, http://links.lww.com/PG9/A76). We also surveyed the frequency of educational activities on CD and pediatrician’s utility and attitudes towards electronic-health technologies (eHealth), such as video calls, as part of CD follow-up care.

#### Survey of Dietitian-led Follow-up

In a 32-question survey, we examined the dietitian-led follow-up practice, including frequency of follow-up and what it comprised of in terms of dietary counseling and adherence assessment, among others (See Supplemental Digital Content 3 Table, http://links.lww.com/PG9/A76). We also surveyed their utility and attitude towards eHealth as part of the dietitian-led follow-up of CD.

### Statistical Methods

Statistical analyses were performed in SPSS version 26 (IBM Corp., USA). Categorical variables were presented as proportions (%), while continuous data were presented as median and interquartile range (IQR). We used the Kruskal-Wallis test to compare variables across subgroups of outpatient clinics organized in VGR according to their geographic location. Statistical significance was defined as p-values less than 0.05.

### Ethics

The study was approved by the Swedish Ethical Review Authority (Dnr: 2020-06107), which waived the requirement of ethical vetting as the study did not collect personal data.

## RESULTS

### Pediatrician-led Follow-up of Coeliac Disease

Fourth-eight pediatricians, representing 18 of 22 pediatric outpatient clinics, responded to the survey. Eight of the respondents were pediatric residents and 40 pediatric specialists, most of whom (n = 33/40 [83%]) defined themselves as general pediatricians, six (15%) were pediatric gastroenterologists, and one (3%) was a pediatric allergist.

#### Follow-up

Nine of 10 (n = 45/48 [94%]) physicians would usually have the first follow-up visit within 6 months from CD diagnosis (Table 1). Thereafter, in case of clinical improvement, the second follow-up visit was by a majority of the respondents (n = 26/48 [54%]) scheduled within 10-12 months from the first visit; however, in children without clinical improvement since diagnosis, respondents would have the second follow-up visit as early as 3-6 months from the first visit (Table 1). Children considered to be in clinical remission were mostly followed yearly until they turned 18 years. The follow-up interval was determined by patient age, symptomatology, and laboratory test results. While a majority of the physicians (n = 25/48 [52%]) reported that quality of life was assessed at each visit, this was entirely assessed through dialogue with the patient, that is, without the use of validated surveys. In contrast, one-third of the physicians (n = 14/48 [29%]) reported that they “never” or “almost never” assessed quality of life during CD follow-up.

**Table 1.** Frequency of pediatrician follow-up of childhood coeliac disease in different clinical scenarios

	First follow-up visit after diagnosis	Second follow-up visit if the disease is in remission	Second follow-up visit if *no* clear improvement	Follow-up intervals when the disease is stable
	n (%)	n (%)	n (%)	n (%)
Pediatricians				
0–3 months	17 (35)	1 (2)	26 (54)	
4–6 months	28 (58)	13 (27)	20 (42)	1 (2)
7–9 months		2 (4)		
10–12 months		26 (54)		15 (34)
13–18 months		1 (2)		16 (36)
19–24 months				11 (25)
Other	3 (6)	5 (10)	2 (4)	1 (2)
Total	48 (100)	48 (100)	48 (100)	44 (100)
Dietitians				
0–3 months	8 (73)			
4–6 months	1 (9)			
10–12 months	1 (9)			
Other	1 (9)			
Total	11 (100)			

Proportion is not summed to 100% due to rounding to integers.

Transition to adult care normally consisted of a referral letter to an adult clinic (Supplemental Digital Content 4 Table, http://links.lww.com/PG9/A76). However, at least 1 in 10 physicians (n = 6/48 [13%]) reported no formal transition to adult care. The existence of a formal transition process to adult care varied considerably (0–100%) between outpatient clinics.

#### Assessment of Dietary Adherence

All pediatricians reported that GFD adherence was virtually always assessed through unstructured interviewing the patient, typically combined with TTG tests, although other serological markers were also occasionally used (Table 2). A follow-up duodenal biopsy was not routinely recommended, but 42% (n = 20/48) of the respondents would consider it in selected cases. Validated questionaries to assess GFD adherence were rarely used by pediatricians. Gluten immunogenic peptides to monitor gluten intake had not been incorporated into clinical practice.

**Table 2.** Assessments of gluten-free dietary adherence, comorbidities and complications during a doctor-led pediatric coeliac clinic

	Frequency of tests used					
	Yes, always	Yes, almost always	Only in case of clinical suspicion	No, rarely	No, never	Row total
	n (%)	n (%)	n (%)	n (%)	n (%)	n (%)
GFD adherence						
Transglutaminase	25 (52)	14 (29)	9 (19)			48 (100)
Endomysium, antibodies			2 (4)	5 (10)	41 (85)	48 (100)
Deamidated gliadin antibodies			5 (10)	6 (13)	37 (77)	48 (100)
Anti-gliadin antibodies			3 (6)	5 (10)	40 (83)	48 (100)
Point-of-care-tests for TTG					48 (100)	48 (100)
Gluten immunogenic peptides					48 (100)	48 (100)
Control biopsy			20 (42)	13 (27)	15 (31)	48 (100)
Vitamins and minerals						
Vitamin D-status	1 (2)	5 (11)	23 (52)	7 (16)	8 (18)	44 (100)
Other vitamins		1 (2)	12 (28)	13 (30)	17 (40)	43 (100)
eg, zinc, magnesium			8 (20)	13 (32)	20 (49)	41 (100)
Comorbidity screening						
Blood counts	4 (9)	9 (20)	25 (54)	8 (17)		46 (100)
Iron levels	1 (2)	4 (9)	36 (80)	4 (9)		45 (100)
Liver function tests	9 (19)	11 (23)	22 (47)	5 (11)		47 (100)
Thyroid function tests	12 (25)	27 (56)	8 (17)	1 (2)		48 (100)
Other blood samples	1 (3)		8 (26)	4 (13)	18 (58)	31 (100)
Hepatitis-B-vaccination			2 (5)	6 (14)	34 (81)	42 (100)
Bone mineral density			3 (7)	7 (16)	33 (77)	43 (100)

Missingness, n = 0–17. Proportion is not summed to 100% due to rounding to integers.

TTG, anti-tissue transglutaminase autoantibodies.

#### Assessment of Associated Diseases and Disease Complications

All pediatricians routinely measured height and weight. Next to TTG, thyroid function tests were the most commonly used blood test. Other laboratory tests and examinations were infrequently used and only ordered as indicated by the patient’s clinical picture (Table 2).

#### Continuing Education

Most pediatricians (n = 26/48 [54%]) received continuing education on CD less often than every 3 years (See Supplemental Digital Content 5 Table, http://links.lww.com/PG9/A76, which describes the frequency of how often the respondents received CD-related educational activities). Despite infrequent learning activities, the self-rated CD knowledge was good, with a median score of 8.0 (IQR = 5.3-8.8) on a scale from 0 to 10, with higher scores indicating better knowledge of CD. However, CD knowledge varied significantly between outpatient clinics across the region (median score ranging from 5.0 [IQR = 3.0-6.5] to 8.0 [7.0-9.0]; *P* value 0.002 for between-groups difference).

#### The Use of E-Health

While the use of eHealth technologies, such as video calls, was rare, the vast majority (n = 38/48 [79%]) of the respondents considered it a good alternative to in-office visits for pediatric CD patients who were in remission (See Supplemental Digital Content 6 Table, http://links.lww.com/PG9/A76, which displays the estimation of how many visits that could be conducted through eHealth technologies).

### The Dietitian-led Follow-up of Pediatric Coeliac Disease

Twelve dietitians from 12 outpatient clinics participated in the survey. The responding dietitians usually met 31-50 coeliac patients annually, and most (n = 7/11 [64%]) had less than 5 years of clinical experience with pediatric CD.

#### Follow-up

Two-thirds of the dietitians (n = 8/11 [73%]) met newly-diagnosed CD patients within 0-3 months from diagnosis (Table 1). After that, the follow-up frequency was determined by patient symptomatology, patient’s request, and clinical status. All responding dietitians believed that a dietitian-led, rather than doctor-led coeliac clinic should be offered to all patients, or at least to selected cases. In contrast to the results from the pediatricians, most dietitians (n = 6/11 [55%]) assessed GFD adherence through a structured interview.

#### Continuing Education and the Use of eHealth

Eight dietitians (n = 8/11 [73%]) reported receiving continuing education more rarely than every 3 years. On a 10-point scale, the median score of CD knowledge was 9.0 (IQR = 6.0-9.0).

While few dietitians had utilized eHealth technologies, such as a video call, in their coeliac clinic, most of them (n = 6/11 [55%]) indicated that it may be appropriately used in as many as 80% of the visits.

## DISCUSSION

This survey found that pediatricians follow children with CD yearly to every second year and that follow-up was similarly performed throughout the Western part of Sweden (VGR region). Although pediatricians assess adherence to the GFD through unstructured interviewing and TTG, dietitians use more structured interviews for that purpose. Except for thyroid function tests, assessments of other comorbidities and complications were only performed in case of clinical suspicion. Both pediatricians and dietitians reported to be knowledgeable about CD, despite infrequent medical education, and they shared a positive attitude towards eHealth as part of CD follow-up.

Although the first ESPGHAN recommendations on CD follow-up in children are expected to be published by 2022, current Swedish guidelines and those from the European Society for the Study of Coeliac Disease (ESsCD) and the American Gastroenterological Association (both based on expert opinions) recommend follow-up yearly to every second year (6,10,11). These guidelines, and other expert reviews on CD management (8,9), also specify the need for continuous dietitian follow-up to succeed in dietary adherence in CD. Overall, our results show that the CD follow-up practice in VGR is in line with these recommendations.

The ESsCD guidelines do not specify how symptoms and dietary adherence should be best assessed during CD follow-up (6). We found that dietary adherence was assessed through unstructured interviewing with the patient and by measures of CD-specific serology tests. While objective and reliable markers of disease, CD-serology has not been developed for disease monitoring during follow-up, and their reported sensitivity to inadvertent gluten exposure is low (3,12). We found that gluten immunogenic peptide measurement has not been introduced in CD follow-up in Sweden, and similar findings were recently reported in a European survey of CD follow-up where a majority (60%) of the respondents worked at academic institutions (13). A duodenal biopsy has previously been assumed as the gold standard for assessing GFD adherence (12). However, with the advent of a non-biopsy approach to CD diagnosis in children (14), this invasive method is neither feasible nor advised to perform other than in carefully selected pediatric patients. Compared with current practice, more widespread use of validated dietary adherence questionnaires would likely provide better adherence assessment that would be comparative over time and between different physicians and dietitians.

Our dietician survey also suggested a positive view for an expanded role of the dietitian in CD follow-up. Dietitian-led coeliac clinics have been shown to provide similar levels of GFD adherence and to be less costly compared to physician-led follow-up (15). Our data do not indicate how a dietitian-led coeliac clinic would be best organized, eg, in collaboration with gastrointestinal expertise.

Recent research has provided new options for CD management and spurred updated recommendations (14,16) on its management that need to be conveyed to clinical practice. It is therefore notable that both pediatricians and dietitians of this study were infrequently educated about the disease. Still, the respondents perceived their knowledge of CD as good, at least in most outpatient clinics. Some clinics reported significantly lower CD expertise suggesting that further education could improve CD follow-up care.

The surveys were sent out during the current COVID-19 pandemic, which likely positively affected the attitude towards eHealth (17), and possibly other remote patient monitoring systems. While few respondents carried out follow-up visits via eHealth, our data indicated that such technologies could be used more frequently during CD follow-up. However, although eHealth is regarded as a potentially cost-effective method for follow-up (18), more research is needed on its utility and effectiveness in CD follow-up care.

A strength of this study is our comprehensive surveys that comprised many different aspects of CD care. The high participation rate, with responses from 18 of 22 outpatient clinics in VGR (2 of 4 non-participating outpatient clinics were small and collaborated closely with clinics that were part of the study) enabled a broad description of the pediatric follow-up practice of CD throughout the region, including both urban and more rural areas. Although unverifiable, our results are therefore likely also representative of follow-up practice of pediatric CD in Sweden in general.

A limitation of this study is that we were unable to examine if the follow-up was carried out according to the reported practice. However, while based on self-reports, the anonymous nature of our survey should have increased the accuracy of the reported clinical practice. Similarly, CD knowledge level was self-rated and not tested. Future research based on patient-reported data or data from patient charts should try to corroborate our findings. While all clinics were part of the same region (VGR, ie, Western Sweden) we lacked data on potential organizational differences across clinics that may explain varying routines for pediatrician- and dietician-led CD follow-up. Finally, we acknowledge that the participation rate was relatively low among dietitians where we received responses from 12 of 22 outpatient clinics; thus, those results should be cautiously interpreted. The low participation rate among dieticians also prevented any formal comparison in their routine for CD follow-up care vs. that of pediatricians.

## CONCLUSION

Pediatric CD follow-up seems largely to be in line with both Swedish and international CD guidelines (6,10,11). However, a number of potential areas of improvement were identified, including a formal transition process to adult healthcare, the use of validated GFD adherence questionnaires, and more frequent educational activities on CD. An expanded role of the dietitian during follow-up was also indicated. Finally, a positive attitude towards eHealth as part of CD follow-up was identified that should be further examined.

## Supplementary Material


