# Supplementary material for: Real-world Follow-up Practice of Children With Coeliac Disease: A Cross-sectional Study From Western Sweden
Source: JPGN Rep. 2022 Mar 17;3(2):e191. doi: 10.1097/PG9.0000000000000191 (PMC10158403; doi:10.1097/PG9.0000000000000191)
Supplement: Supplementary file 1 [file pg9-3-e191-s001.pdf]

SUPPLEMENTAL DIGITAL CONTENT

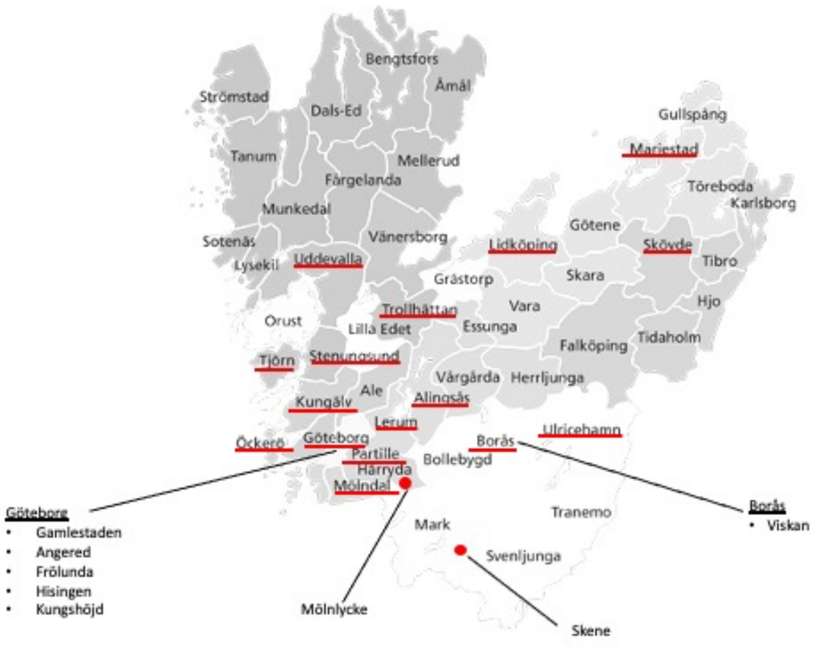

Figure, Supplemental Digital Content 1. Map over Region Västra Götaland. All underlined cities have one or more paediatric outpatient clinics. Two out of four non-participating outpatient clinics were small and collaborated closely with clinics that were part of the study. Responding paediatricians per clinic: Gamlestaden 2; Angered 1; Alingsås 1; Frölunda 3; Hisingen 3; Kungshöjd 6; Kungälv 3; Lerum 1; Mölndal 2; Mölnlycke 3; Partille 3; Skene 1; Stenungsund 2; Skövde 2; Mariestad 1; Lidköping 2; Trollhättan 9; Uddevalla 3.

Table, Supplemental Digital Content 2. Questions and response alternatives in survey of paediatrician-led follow-up (the number of questions detailed below do not sum up to 52 since layout for specific questions/response alternatives differ from what were used in the web-based survey).

**Question**	**Response alternative**
Which paediatric outpatient clinic do you work at (Name/geographic location)?	• Gamlestaden<br>• Angered<br>• Alingsås<br>• Frölunda<br>• Hisingen<br>• Kungshöjd<br>• Kungälv<br>• Lerum<br>• Mölndal<br>• Mölnlycke<br>• Partille<br>• Skene<br>• Stenungsund<br>• Tjörn<br>• Ulricehamn<br>• Viskan<br>• Öckerö<br>• Skövde<br>• Mariestad<br>• Lidköping<br>• Trollhättan<br>• Uddevalla
What is your level of employment at the paediatric outpatient clinic?	• 25%<br>• 50%<br>• 75%<br>• 100%
Are you a specialist doctor?	• Yes<br>• No
If you are a specialist doctor, which of the following alternatives do best describe your specialty?	• Paediatrician (general)<br>• Paediatrician specializing in gastroenterology<br>• Paediatric allergist<br>• Other
For how many years have you been a specialist doctor?	• <5 years<br>• 5-9 years<br>• 10-14 years<br>• 15 years or more
Do you currently care for patients with coeliac disease?	• Yes<br>• No
For how many years have you been managing follow-up care of coeliac disease?	• <5 years<br>• 5-9 years<br>• 10-14 years<br>• 15 years or more
How many celiac patients do you annually meet for follow ups?	• 1-10 patients<br>• 11-30 patients<br>• 31-50 patients<br>• 50 patients or more
How regularly do you measure WEIGHT at follow-up visits for coeliac disease?	• Always<br>• Almost always<br>• Sometimes<br>• Almost never<br>• Never

How regularly do you measure LENGHT at follow-up visits for coeliac disease?	• Always<br>• Almost always<br>• Sometimes<br>• Almost never<br>• Never	
How regularly do you measure OTHER anthropometrics at follow-up visits for coeliac disease?	• Always<br>• Almost always<br>• Sometimes<br>• Almost never<br>• Never	
If you answered "other" in the previous question; please specify what you are measuring.		
During follow-up visits for coeliac disease, how regularly do you measure quality of life?	• Always<br>• Almost always<br>• Sometimes<br>• Almost never<br>• Never	
If quality of life is measured, what method do you use?		
During follow-up visits for coeliac disease, how regularly do you measure adherence to the gluten-free diet?	• Always<br>• Almost always<br>• Sometimes<br>• Almost never<br>• Never	
If adherence to the gluten-free diet is measured, what method(s) do you use?	• Dialogue with the patient<br>• Structured interviewing<br>• A rating scale (unspecified)<br>• Antibody measurement (unspecified)<br>• Biopsy<br>• Refers to dietitian	
What laboratory tests/investigations are part of your follow-up visits for coeliac disease?<br><br>For each investigation listed, indicate at what frequency/situation they are being used.	• Transglutaminase, TGA<br>• Endomysium antibodies, EMA<br>• Deamidated gliadin, dGP<br>• Anti-gliadin antibodies, AGA<br>• "Point-of-care test for TGA"<br>• Gluten immunogenic peptide (GIP) in urine or faeces<br>• Referral for control biopsy	• Yes, always (at each visit)<br>• Yes, almost always<br>• Yes, regularly performed regardless of clinical picture<br>• Yes, but only when clinically indicated/specific situations<br>• No, almost never<br>• No, never
If you refer for control biopsy (answered YES above), indicate		

in what situations/cases you recommend it/refer for that.		
What other laboratory tests/investigations/procedures are part of your follow-up visits for coeliac disease?<br><br>For each test/investigation/procedure listed, indicate at what frequency/situation they are being used.	• Blood counts<br>• Iron levels<br>• Liver function tests<br>• Hepatitis B-vaccination<br>• Vitamin D (25-OH-D)-status<br>• Other vitamins<br>• Trace elements (e.g. zinc, magnesium)<br>• Thyroid function tests<br>• Bone density (DXA)<br>• Other blood samples	• Yes, always (at each visit)<br>• Yes, almost always<br>• Yes, regularly performed regardless of clinical picture<br>• Yes, but only when clinically indicated/specific situations<br>• No, almost never<br>• No, never<br>• No, not relevant
<u>If you answered yes to the question on "other vitamins"</u>, please specify what vitamins.		
Do you recommend any of the following supplements at your follow ups for coeliac disease?<br><br>For each supplement, indicate at what frequency/situation they are being used.	• Iron supplements<br>• Vitamin D<br>• Calcium<br>• Multivitamins (unspecified)	• Yes, always (at each visit)<br>• Yes, almost always<br>• Yes, regularly regardless of clinical picture<br>• Yes, but only when clinically indicated/specific situations<br>• No, almost never<br>• No, never
Do you have a dietitian available at your paediatric outpatient clinic?	• Yes<br>• No	
Estimate what proportion of your coeliac patients who also sees a dietitian as part of their follow-up for the disease?	• 0%<br>• 10%<br>• 20%<br>• 30%<br>• 40%<br>• 50%	• 60%<br>• 70%<br>• 80%<br>• 90%<br>• 100%
Do you have a psychologist available at your paediatric outpatient clinic?	• Yes<br>• No	
Estimate what proportion of your coeliac patients who also sees a psychologist as part of their follow-up for the disease?	• 0%<br>• 10%<br>• 20%	• 60%<br>• 70%<br>• 80%

	• 30%<br>• 40%<br>• 50%	• 90%<br>• 100%
Estimate what proportion of your coeliac patients whom by you been recommended membership in a coeliac disease support group / Patient Association?	• 0%<br>• 10%<br>• 20%<br>• 30%<br>• 40%<br>• 50%	• 60%<br>• 70%<br>• 80%<br>• 90%<br>• 100%
Estimate what proportion of your patients/their families that are members in a coeliac disease support group / Patient Association?	• 0%<br>• 10%<br>• 20%<br>• 30%<br>• 40%<br>• 50%	• 60%<br>• 70%<br>• 80%<br>• 90%<br>• 100%
How soon after coeliac disease diagnosis do you usually schedule your first follow-up visit?	• 0-3 months<br>• 4-6 months<br>• 7-9 months<br>• 10-12 months<br>• 13-18 months	• 19-24 months<br>• More than 24 months<br>• No regularly visits are planned<br>• Other
What determines the frequency of your follow ups? (you may choose several options)	• Symptomatology<br>• The value of laboratory results (e.g. transglutaminase, TGA)<br>• Patient's age<br>• Same schedule/priority for all patients<br>• Other reason	
<u>If you selected "other reason" in the previous question;</u> please specify.	•	
In the case of clear improvement in symptoms and/or laboratory tests (incl. normalisation / decreasing transglutaminase levels) by the time of the first follow-up visit, how soon after do you usually schedule the next visit?	• 0-3 months<br>• 4-6 months<br>• 7-9 months<br>• 10-12 months<br>• 13-18 months	• 19-24 months<br>• More than 24 months<br>• No regularly visits are planned<br>• Other
In the case on NO clear improvement in symptoms and/or laboratory tests by the time of the first follow-up visit, how soon after do you schedule the next visit?	• 0-3 months<br>• 4-6 months<br>• 7-9 months<br>• 10-12 months<br>• 13-18 months	• 19-24 months<br>• More than 24 months<br>• No regularly visits are planned<br>• Other
When the patient's clinical condition is good, at what frequency (on average) do you schedule follow-up visits for	• 0-3 months<br>• 4-6 months<br>• 7-9 months<br>• 10-12 months<br>• 13-18 months	• 19-24 months<br>• More than 24 months<br>• No regularly visits are planned<br>• Other

coeliac disease? Answer in time intervals.	
Do you have more frequent follow ups during adolescence?	• Yes, most often or almost always<br>• Yes, sometimes, depending on the patient's situation<br>• No, never
On average, how many minutes are scheduled per visit for coeliac disease take?	
On a scale, how satisfied are you with the time allocated per visit?	Scale ranging from 1 to 7: 1 = very dissatisfied, 7 = very satisfied
Do screen/recommend coeliac disease screening of family members to coeliac patients?	• Yes, always<br>• Yes, sometimes<br>• No, almost never<br>• No, never
How is the typical transition process to adult care for children with coeliac disease?	• Joint visit with adult unit/physician<br>• Through specific protocol<br>• Through a referral letter to adult unit/physician<br>• No formal transition<br>• Other
If you answered "other" to the previous question; please specify.	
On a scale, how satisfied are you with the current transition process to the adult care?	Scale ranging from 1 to 7: 1 = very dissatisfied, 7 = very satisfied
Estimate what proportion of your coeliac patients who are transferred to adult care?	• 0%<br>• 10%<br>• 20%<br>• 30%<br>• 40%<br>• 50%<br>• 60%<br>• 70%<br>• 80%<br>• 90%<br>• 100%
How frequently do you receive continuing medical education on coeliac disease?	• Every year<br>• Every two years<br>• Every three years<br>• More rarely (than every three years)<br>• Other
On a scale, how well do you agree with the following statement: “I feel that I have sufficient knowledge about coeliac disease and its follow-up practice”.	Scale ranging from 0 to 10 where ten is indicated by “I have sufficient knowledge about coeliac disease and its follow-up” and zero is indicated by “I do NOT have sufficient knowledge about coeliac disease and its follow-up”.
Have you for **any reason** over the past 6 months performed an online consultation (video call)?	• 0<br>• 1<br>• 2<br>• 3<br>• 4<br>• 5<br>• More than 5
Have you over the past 6 months had an online consultation	• 0<br>• 1<br>• 4<br>• 5

(video call) specifically for **coeliac disease**?	• 2<br>• 3<br>• More than 5
In the case of a good clinical condition after coeliac disease diagnosis, to what extent do you think routine checks could be done through online consultations/digital care?	• 0%<br>• 10%<br>• 20%<br>• 30%<br>• 40%<br>• 50%<br>• 60%<br>• 70%<br>• 80%<br>• 90%<br>• 100%
To what extent do you think (extra) visits prompted by symptoms of coeliac disease could be done through online consultations/digital care?	• 0%<br>• 10%<br>• 20%<br>• 30%<br>• 40%<br>• 50%<br>• 60%<br>• 70%<br>• 80%<br>• 90%<br>• 100%
In the case of remote care, what alternative would prefer the most for check-ups for coeliac disease?	• Phone call<br>• Texting<br>• Chat<br>• Video call<br>• Mail<br>• Other
If you answered "other", please specify.	
Overall, how do you value/regard **physician-led** digital follow-up visits for coeliac disease?	• Very good<br>• Fairly good<br>• Fairly bad<br>• Very bad
Overall, how do you value/regard **dietican-led** digital follow-up visits for coeliac disease?	• Very good<br>• Fairly good<br>• Fairly bad<br>• Very bad
To what extent do you agree with the following statements on digital follow-up visits for coeliac disease?	• Better quality of care<br>• Deteriorated patient safety<br>• Deteriorated personal contact<br>• Over-utilization of the resources of paediatric clinics<br>• The patients have improved access to care<br>• More effective care overall<br><br>Scale ranging from 1 to 7, 1 = do not agree at all, 7 = completely agree
Estimate the proportion of coeliac patients to whom you recommend use of mHealth technologies (e.g., apps) AT HOME to facilitate their adherence to the gluten-free diet?	• 0%<br>• 10%<br>• 20%<br>• 30%<br>• 40%<br>• 50%<br>• 60%<br>• 70%<br>• 80%<br>• 90%<br>• 100%
Estimate the proportion of coeliac patients to whom you	• 0%<br>• 10%<br>• 60%<br>• 70%

recommend use of mHealth technologies (e.g., apps) OUTSIDE THE HOME (at friends, in school etc) to facilitate their adherence to the gluten-free diet?	• 20%<br>• 30%<br>• 40%<br>• 50%	• 80%<br>• 90%<br>• 100%
Estimate the proportion of coeliac patients to whom you recommend use of mHealth technologies (e.g., apps) to retrieve INFORMATION on the disease/symptoms?	• 0%<br>• 10%<br>• 20%<br>• 30%<br>• 40%<br>• 50%	• 60%<br>• 70%<br>• 80%<br>• 90%<br>• 100%
Finally, if there is some aspect of coeliac follow-up care that this survey did not cover, or an aspect that you think should be improved in coeliac follow ups, you may indicate that here.		

Table, Supplemental Digital Content 3. Questions and response alternatives in survey of dietician-led follow-up (the layout below for specific questions/response alternatives differ from what were used in the web-based survey).

Question	Response alternative	
Which paediatric outpatient clinic do you work at (Name/geographic location)?	• Gamlestaden<br>• Angered<br>• Alingsås<br>• Frölunda<br>• Hisingen<br>• Kungshöjd<br>• Kungälv<br>• Lerum<br>• Mölndal<br>• Mölnlycke<br>• Partille	• Skene<br>• Stenungsund<br>• Tjörn<br>• Ulricehamn<br>• Viskan<br>• Öckerö<br>• Skövde<br>• Mariestad<br>• Lidköping<br>• Trollhättan<br>• Uddevalla
What is your level of employment at the paediatric outpatient clinic?	• 25%<br>• 50%<br>• 75%<br>• 100%	
Do you currently care for patients with coeliac disease?	• Yes<br>• No	
For how many years have you been managing follow-up care of coeliac disease?	• <5 years<br>• 5-9 years<br>• 10-14 years<br>• 15 years or more	
How many celiac patients do you annually meet for follow ups?	• 1-10 patients<br>• 11-30 patients<br>• 31-50 patients<br>• 50 patients or more	
During follow-up visits for coeliac disease, do you regularly measure adherence to the gluten-free diet?	• Always<br>• Almost always<br>• Sometimes<br>• Almost never<br>• Never	
If adherence to the gluten-free diet is measured, what method(s) do you use?	• Dialogue with the patient<br>• Structured interviewing<br>• A rating scale (unspecified)<br>• Other	
Do you recommend any of the following supplements at your follow ups for coeliac disease?<br><br>For each supplement, indicate at what frequency/situation they are being used.	• Iron supplements<br>• Vitamin D<br>• Calcium<br>• Multivitamins (unspecified)	• Yes, always (at each visit)<br>• Yes, almost always<br>• Yes, regularly regardless of clinical picture<br>• Yes, but only when clinically indicated/specific situations

		• No, almost never<br>• No, never
Estimate what proportion of your coeliac patients whom by you been recommended membership in a coeliac disease support group / Patient Association?	• 0%<br>• 10%<br>• 20%<br>• 30%<br>• 40%<br>• 50%	• 60%<br>• 70%<br>• 80%<br>• 90%<br>• 100%
Estimate what proportion of your patients/their families that are members in a coeliac disease support group / Patient Association?	• 0%<br>• 10%<br>• 20%<br>• 30%<br>• 40%<br>• 50%	• 60%<br>• 70%<br>• 80%<br>• 90%<br>• 100%
How soon after coeliac disease diagnosis do you usually schedule your (dietician) first follow-up visit?	• 0-3 months<br>• 4-6 months<br>• 7-9 months<br>• 10-12 months<br>• 13-18 months	• 19-24 months<br>• More than 24 months<br>• No regularly visits are planned<br>• Other
What determines the frequency of your (dietician) follow ups? (you may choose several options)	• Symptomatology<br>• Patient's age<br>• Same schedule/priority for all patients<br>• Patient's preference<br>• Other reason	
<u>If you selected "other reason" in the previous question;</u> please specify.	•	
Do you have more frequent follow ups during adolescence?	• Yes, most often or almost always<br>• Yes, sometimes, depending on the patient's situation<br>• No, never	
Do you have group consultations/education sessions on gluten-free diet for coeliac patients?	• Yes<br>• No	
On average, how many minutes are scheduled per visit for coeliac disease?		
On a scale, how satisfied are you with the collaboration between you and the patient's physician?	Scale ranging from 0 to 10: 0 = very dissatisfied, 10 = very satisfied	
Could the follow-up of coeliac patients be done with dietitian visits instead of physician visits (i.e., replace such visits)?	• Yes, always<br>• Yes, in selected cases<br>• No, never	
How is your work developing around patients with coeliac disease? What challenges do you		

face in today's work with these patients?	
How frequently do you receive continuing medical education on coeliac disease?	• Every year<br>• Every two years<br>• Every three years<br>• More rarely (than every three years)<br>• Other
What type of education do you get about coeliac disease?	
On a scale, how well do you agree with the following statement: “I feel that I have sufficient knowledge about coeliac disease and its follow-up practice”.	Scale ranging from 0 to 10 where ten is indicated by “I have sufficient knowledge about coeliac disease and its follow-up” and zero is indicated by “I do NOT have sufficient knowledge about coeliac disease and its follow-up”.
Have you for **any reason** over the past 6 months performed an online consultation (video call)?	• 0<br>• 1<br>• 2<br>• 3<br>• 4<br>• 5<br>• More than 5
Have you over the past 6 months had an online consultation (video call) specifically for **coeliac disease**?	• 0<br>• 1<br>• 2<br>• 3<br>• 4<br>• 5<br>• More than 5
To what extent do you think routine checks/dietician counselling could be done through online consultations/digital care?	• 0%<br>• 10%<br>• 20%<br>• 30%<br>• 40%<br>• 50%<br>• 60%<br>• 70%<br>• 80%<br>• 90%<br>• 100%
In the case of remote care, what alternative would prefer the most for check-ups for coeliac disease?	• Phone call<br>• Texting<br>• Chat<br>• Video call<br>• Mail<br>• Other
If you answered “other”, please specify.	
Overall, how do you value/regard **dietican-led** digital follow-up visits for coeliac disease?	• Very good<br>• Fairly good<br>• Fairly bad<br>• Very bad
To what extent do you agree with the following statements on digital follow-up visits for coeliac disease?	• Better quality of care<br>• Deteriorated patient safety<br>• Deteriorated personal contact<br>• Over-utilization of the resources of paediatric clinics<br>• The patients have improved access to care<br>• More effective care overall

	Scale ranging from 1 to 7, 1 = do not agree at all, 7 = completely agree
Estimate the proportion of coeliac patients to whom you recommend use of mHealth technologies (e.g., apps) AT HOME to facilitate their adherence to the gluten-free diet?	• 0%<br>• 10%<br>• 20%<br>• 30%<br>• 40%<br>• 50%<br>• 60%<br>• 70%<br>• 80%<br>• 90%<br>• 100%
Estimate the proportion of coeliac patients to whom you recommend use of mHealth technologies (e.g., apps) OUTSIDE THE HOME (at friends, in school etc) to facilitate their adherence to the gluten-free diet?	• 0%<br>• 10%<br>• 20%<br>• 30%<br>• 40%<br>• 50%<br>• 60%<br>• 70%<br>• 80%<br>• 90%<br>• 100%
Estimate the proportion of coeliac patients to whom you recommend use of mHealth technologies (e.g., apps) to retrieve INFORMATION on the disease/symptoms?	• 0%<br>• 10%<br>• 20%<br>• 30%<br>• 40%<br>• 50%<br>• 60%<br>• 70%<br>• 80%<br>• 90%<br>• 100%
Finally, if there is some aspect of coeliac follow-up care/dietician counselling that this survey did not cover, or an aspect that you think should be improved in coeliac follow ups, you may indicate that here.	

Table, Supplemental Digital Content 4. Surveyed transition options used by paediatricians.

	**No formal transition**	**Joint visit with adult unit/physician**	**Through specific protocol**	**Through referral letter to adult unit/physician**	**Other option** *
	Respondents (%)	*Respondents (%)*	*Respondents (%)*	*Respondents (%)*	*Respondents (%)*
Yes	6 (13)			40 (83)	4 (8)
No	42 (88)	48 (100)	48 (100)	8 (17)	44 (92)
Total	48 (100)	48 (100)	48 (100)	48 (100)	48 (100)

*Open-ended response alternative that included the promotion of “self-referral” for adult care.

Table, Supplemental digital content 5. Frequency of how often the respondents receive continuing education.

	**Paediatricians**	**Dietitians**
	n (%)	*n (%)*
Every year	4 (8)	2 (18)
Every two years	6 (13)	
Every three years	7 (14)	1 (9)
More rarely	26 (54)	8 (73)
Other	5 (10)	
Total	48 (100)	11 (100)

Table, Supplemental Digital Content 6. Estimate of how many coeliac disease visits could be conducted with the use of eHealth technologies.

	**Paediatricians**	**Dietitians**
	n (%)	*n (%)*
10%	1 (2)	
20%	2 (4)	
30%	6 (13)	
40%	1 (2)	
50%	13 (27)	
60%		1 (9)
70%	9 (19)	2 (18)
80%	10 (21)	6 (55)
90%	4 (8)	1 (9)
100%	2 (4)	1 (9)
Total	48 (100)	11 (100)
